# “Piggy-Backing” on Diagnostic Platforms Brings Hope to Neglected Diseases: The Case of Sleeping Sickness

**DOI:** 10.1371/journal.pntd.0000715

**Published:** 2010-05-25

**Authors:** Joseph Mathu Ndung'u, Sylvain Bieler, Giorgio Roscigno

**Affiliations:** Foundation for Innovative New Diagnostics (FIND), Geneva, Switzerland; New York University School of Medicine, United States of America

Neglected infectious diseases (NIDs) attract little interest from commercial companies that invest in diagnostics and therapeutics, mainly because the people that they affect are amongst the poorest in the world, who cannot afford to pay for them. Many commercial companies shy away from manufacturing diagnostic tests for NIDs because a return on investment is not usually guaranteed.

It is therefore not surprising that for a disease such as human African trypanosomiasis (HAT), or sleeping sickness, no diagnostic test has ever been manufactured under full registration by any regulatory agency. Tests that are available today are produced by academic institutions, with no guarantee that good manufacturing practice for *in-vitro* diagnostics (GMP-IVD) is adhered to. The card agglutination test for trypanosomiasis (CATT), developed in 1978, is the primary screening tool used in areas where *Trypanosoma brucei gambiense* is endemic [1]. Detection of antibodies against trypanosomes using CATT is a sensitive indicator of infection. However, in populations undergoing screening, where prevalence of the disease is usually below 2% and specificity of the CATT test is around 95%, a large number of positive results turn out to be false-positives, and the positive predictive value of the test is not good enough for it to be used on its own to guide treatment. The test is manufactured using whole *T. b. gambiense* organisms recovered from infected laboratory animals in a complex and risky process, has inferior sensitivity in some disease foci, and can be performed only by trained personnel. Furthermore, it is incapable of differentiating between active and cured infections, as antibodies tend to stay in the blood for prolonged periods after patients have been cured [2], [3]. What is worse, no similar test is available for *T. b. rhodesiense* infection.

Until now, no successful attempt has been made to transform the CATT into a single-format lateral flow test (LFT), which would make it more accessible to diagnostics facilities. This could again be because a LFT for HAT provides little promise for a return on investment, especially if it is to be delivered at a price that is affordable to the public sector in endemic countries. Yet for diseases that are comparatively more attractive, such as tuberculosis (TB), HIV, malaria, and avian and swine flu, there has been more commercial interest. In the late 1990s, intense lobbying by endemic countries, the World Health Organization (WHO) and the international community resulted in a paradigm shift, when at the beginning of this decade, the pharmaceutical industry agreed to provide free drugs for HAT, preventing a potentially embarrassing situation [4]. However, this goodwill could not be extended to diagnostics as no company was manufacturing any tests for HAT.

**Figure 1:**
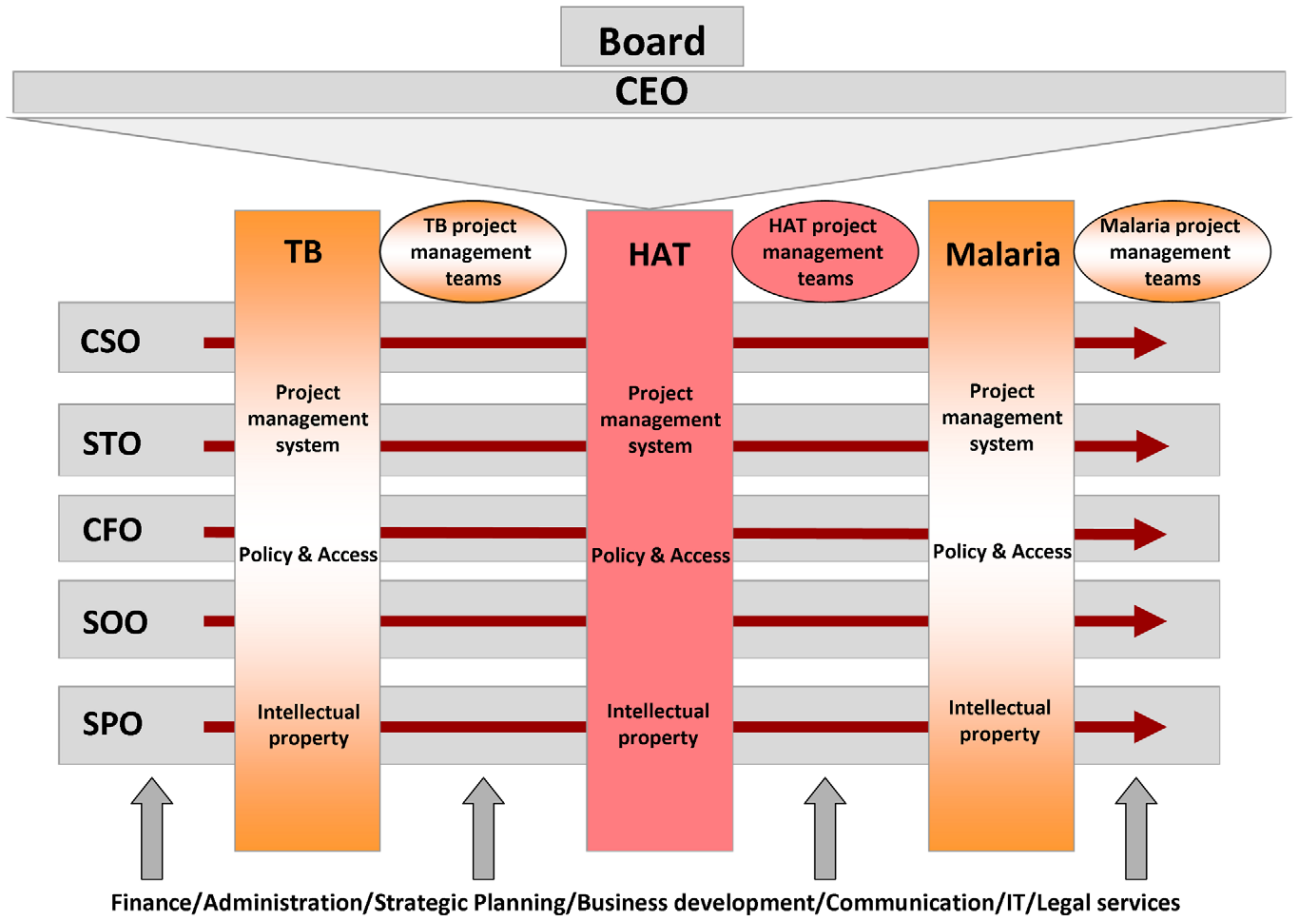
The management structure of FIND. Diagnostic programmes for various diseases operate as independent business units, supported by expertise that cuts across the programmes. SAC: scientific advisory committee, CSO: chief scientific officer, STO: senior technology officer, CFO: chief finance officer, SOO: senior operations officer, SPO: senior policy officer.

**Figure 2:**
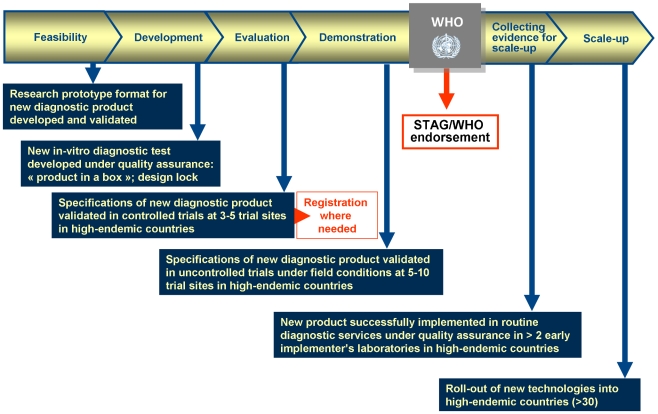
Phases in the diagnostics development pipeline that are implemented by FIND. A product must meet pre-determined criteria to advance from one stage to the next.

A private foundation in Switzerland, the Foundation for Innovative New Diagnostics (FIND), has devised a novel approach towards development of diagnostic tests for NIDs that is generating a lot of interest in industry. FIND, established in 2003, supports the development of diagnostic tests for diseases of poverty, including TB, HAT, and malaria. The unique management structure of FIND comprises diagnostics programmes that exist as independent vertical business units, supported by expertise that cuts across them (Figure 1). A hallmark of FIND's style of project management includes the structuring of product development, evaluation, demonstration and implementation into phases that are well defined, with deliverables and milestones that must be met before products can advance in the pipeline and receive further investment (Figure 2). The rigor of FIND's project management has been recognised through certification for ISO 13485:2003 and 9001:2008, standards that are customary for *in vitro* diagnostics (IVD) manufacturing companies, yet are a rare achievement for a non-profit organisation.

During the first six years of existence, FIND focused its efforts on a diagnostics development approach that seeks technology platforms that are applicable to more than one disease, and used this knowledge to leverage technology development companies to include NIDs in such platforms. Diagnostic products that have passed through development, evaluation, and demonstration trials are integrated into the public health sectors of target countries in partnerships that ensure their sustainable implementation. This has enabled FIND to create a network of partners spanning the entire diagnostics development pipeline, from discovery to implementation [5]. Leveraging its contribution to the collaborations that are established during the product development process, FIND negotiates access strategies that guarantee sustained availability of high-quality tests at affordable prices for the public and non-profit private healthcare sectors. It does this through a laboratory support programme that provides an excellent opportunity to strengthen capacity for diagnosis of NIDs by ensuring introduction, adaptation, and adoption of the most appropriate diagnostic technologies into an integrated laboratory network.

**Figure 3:**
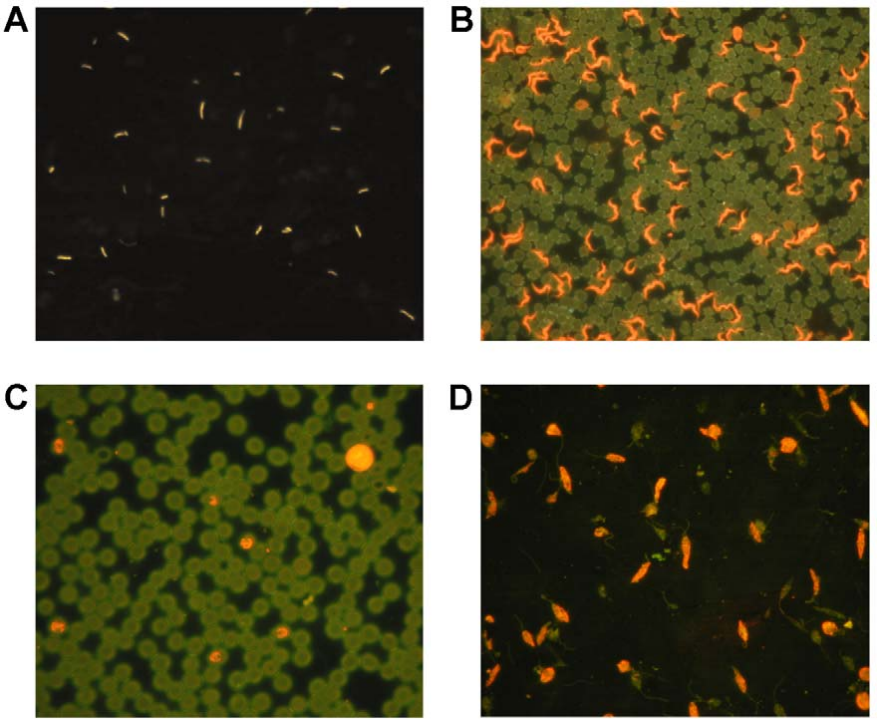
The Primo Star iLED microscope can be used to visualize various pathogens. (A) Tubercle bacilli stained with auramine O (*courtesy of CDC*). (B) A thin blood smear stained with acridine orange showing trypanosomes (orange) alongside red blood cells (green) (*courtesy of Zeiss*). (C) Malaria parasites (small orange structures) inside red blood cells (green) of a baboon experimentally infected with *Plasmodium knowlesi* and stained with acridine orange. White blood cells also stain orange (*blood smear courtesy of Dr. Maina Ngotho*, *Institute of Primate Research*, *Nairobi*). (D) Cultured *Leishmania donovani* promastigotes (orange with flagella) stained with acridine orange (*slide courtesy of Dr. Maina Ngotho*).

Two FIND-supported technology platforms that are applicable to more than one disease have completed development and are now undergoing evaluation for HAT. The first, a light-emitting diode (LED)–based fluorescence microscope developed for TB by FIND and Zeiss has become an excellent tool for parasite demonstration in HAT, and only required evaluation studies to prove its worth [6]. Besides TB and HAT, the microscope has great potential for other indications such as malaria and leishmaniasis (Figure 3). It is robust, affordable, uses LED bulbs with a lifespan of more than 10,000 hours, and does not require a dark room. Since the bulbs use very little energy, the microscope can be operated using solar power, making it easy to use in remote rural settings such as those where these diseases occur. The microscope has been successfully evaluated for HAT in laboratories in Uganda and the Democratic Republic of the Congo (DRC), using acridine orange (AO) as the label [6]. Furthermore, the AO staining procedure is faster than Giemsa (3 versus 45 minutes of incubation).

The number of trypanosomes in the blood of HAT patients is usually low (especially *T. b. gambiense*), and various methods are used to concentrate the parasites in order to see them under a microscope. FIND has been working with scientists at Makerere University, Uganda, and have overcome this problem by performing selective lysis of the red cells in blood samples using ammonium chloride or commercial lysis buffers, without affecting the integrity of parasites [7]. When the lysed samples are centrifuged and the sediment is used to prepare smears, the sensitivity of LED fluorescence microscopy is greatly improved. Parasite concentration by lysis of red cells has a number of advantages over standard parasitological methods used to concentrate trypanosomes: it is a simple and fast technique, no cold chain is required, and large volumes of blood (>5 ml) can be lysed to enhance the sensitivity of trypanosome detection. A combination of this method with LED fluorescence microscopy has great potential for inclusion into the HAT diagnostic algorithm. Indeed, clinical evaluation of this method is set to start at several sites in the DRC and Uganda in 2010. Sustainable manufacture of the LED microscope is guaranteed, because diseases such as TB will provide the market.

**Figure 4:**
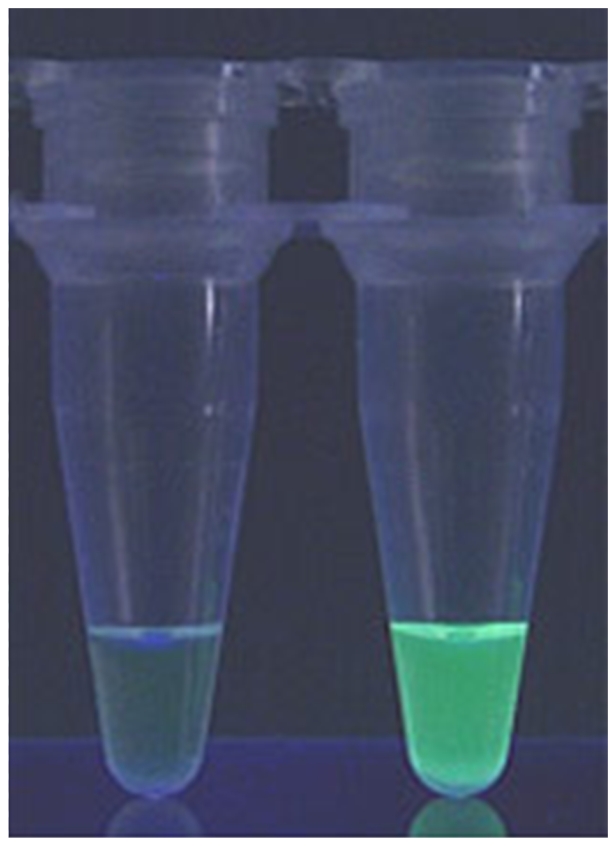
The result of a LAMP test is determined by visual inspection of reaction tubes. Positive samples exhibit bright green fluorescence (right) and are thus easily distinguished from negative ones (left) (*courtesy of Eiken Chemical*).

The second technology, loop-mediated isothermal amplification (LAMP) of DNA, is a simple molecular method developed by Eiken Chemical in Japan. The LAMP test is a novel strategy for DNA amplification that relies on the auto-cycling strand displacement synthesis of DNA by *Bst* DNA polymerase under isothermal conditions (60–65°C) [8]. Since LAMP is carried out at a constant temperature, a simple incubator such as a water bath or heating block is sufficient for DNA amplification. The reaction shows high tolerance to biological products, such that DNA extraction is not necessary. The technique uses a set of six primers that recognise eight sections of target DNA. Simultaneous synthesis of DNA by multiple primers makes LAMP highly sensitive and increases specificity, efficiency, and rapidity. The results of a test can be inspected visually by the addition of the fluorescent dye SYBR Green 1 or calcein (Figure 4), or by measurement of turbidity derived from a precipitate of magnesium pyrophosphate in the reaction mixture.

FIND and its academic partners, Murdoch and Obihiro Universities, have successfully developed a LAMP test for sub-genus *Trypanozoon* using the random insertion mobile element (RIME) sequences [9] and one for *T. b. rhodesiense* based on the serum resistance associated (SRA) gene [10]. Meanwhile, FIND had been working with Eiken on a LAMP test for TB long before they started the HAT programme [11], and has taken advantage of this relationship with Eiken to include the LAMP test for HAT in this diagnostic platform. Evaluation of manufactured LAMP tests for HAT using blood as the starting material is to be carried out in experimental and clinical settings in 2010. Further studies to determine the feasibility of using saliva or urine as the starting sample will also be carried out.

The LAMP test can be performed by staff with minimal experience in molecular biology. Given its high sensitivity, specificity, speed, and ease of use, LAMP could become a good test for field diagnosis of HAT and confirmation of cure in sub-Saharan Africa, where facilities are limited. FIND is working with the Institute of Primate Research (IPR) in Nairobi, Kenya, to determine the feasibility of using this method to confirm cure after successful treatment, and predict relapses in case of treatment failure [12]. Its application as a test of cure will however depend on the rate at which DNA from dead parasites is cleared from a host after treatment. The test could also be useful for epidemiological studies and disease elimination programmes. It also appears that with a little more effort, LAMP tests for Buruli ulcer, Chagas disease, and leishmaniasis, other NIDs that FIND has taken an interest in, could be included on the same platform, whose commercial development targets diseases such as TB and malaria. This platform has therefore provided a good opportunity to diagnose several diseases from the same sample.

The lateral flow test (LFT) provides yet another platform that is widely used in indications such as pregnancy, malaria, etc. In yet another first for FIND, an LFT for screening for HAT could soon be available. An initiative spearheaded by FIND has been screening candidate antigens for their potential in detecting both *T. b. gambiense* and *T. b. rhodesiense*, to be used for developing a specific and sensitive antibody detection LFT [13]. The test will be developed at minimal additional cost in a new partnership between FIND and Standard Diagnostics, Republic of Korea, a commercial company that has become a global leader in development of IVDs for infectious diseases.

The initiatives described here will result in novel tests for HAT that are more sensitive and specific, and are easier to use than those that are currently available. Application of the tests could lead to an acceleration of the present efforts in surveillance and control of the disease. Such tools will also be invaluable in setting up better recruitment of patients and confirmation of cure in clinical studies by pharma and other organizations involved in compound development for HAT.

While FIND may have devised an innovative approach to solve the problem of technology development by investing in platforms that apply to commercially attractive diseases and to “piggy-back” the NIDs on them, the challenge that remains is to increase the investment in strategies that will make these diagnostics accessible, so that they can easily reach the “neglected people” at little or no cost.
